# Chronic diseases multi-morbidity among adult patients at Hawassa University Comprehensive Specialized Hospital

**DOI:** 10.1186/s12889-018-5264-5

**Published:** 2018-03-14

**Authors:** Endrias Markos Woldesemayat, Andargachew Kassa, Taye Gari, Mesay Hailu Dangisso

**Affiliations:** 1 Faculty of Medicine, Centre for International Health, University of Bergen, Bergen, Norway; 2 Hawassa University, School of Public Health, P.O.Box 1352, Hawassa, Ethiopia; 3 Hawassa University, School of Nursing, Hawassa, Ethiopia

**Keywords:** Chronic diseases, Multi-morbidity, Hawassa university

## Abstract

**Background:**

Non-communicable chronic diseases (NCCDs) multi-morbidity is becoming one of the public health problems in Ethiopia. The objective of this study was to describe the prevalence of NCCDs and multi-morbidity among adult patients at Hawassa University Comprehensive Specialized Hospital (HUCSH).

**Methods:**

Between January and February 2016, a cross-sectional study was carried out among patients aged ⩾ 18 years attending the outpatient department of the hospital. Trained nurses interviewed patients and reviewed medical records. Multi-morbidity was defined as the coexistence of two or more NCCDs in an individual.

**Results:**

Two hundred twenty seven (55.2%) of the respondents had at least one of the NCCDs and 73 (17.8%) of them had multi-morbidity. The commonest diseases that affected the patients were diseases of the musculoskeletal system. The risk of having NCCDs was highest among patients aged above 44 years (Adjusted odds ratio (AOR) = 2.7, 95% CI 1.5–4.8). Non educated patients (AOR = 1.7, 95% CI 1.0–2.7) and patients with high household income (AOR = 1.6, 95% CI 1.0–2.5) and patients with a body mass index (BMI) of at least 25 (AOR = 2.0, 95% CI 1.1–3.7) had higher odds of having NCCDs. Highest odds of multi-morbidity was observed among patients aged above 44 years (AOR = 4.4, 95% CI 2.2–8.8).

**Conclusion:**

The prevalence of NCCDs and multi-morbidity among the study population was high. Identifying and addressing modifiable risk factors; screening, treatment and follow-up of patients with NCCDs could help in reducing the burden of NCCDs multi-morbidity and its effect.

**Electronic supplementary material:**

The online version of this article (10.1186/s12889-018-5264-5) contains supplementary material, which is available to authorized users.

## Background

Globally, every year, non-communicable chronic diseases (NCCDs) cause more than 35 million deaths [1]. Of this, 80% deaths occur in low and middle income countries including Ethiopia [1, 2]. The diseases affect any organ of the body and have immense consequence on population health [3]. Sometimes they are considered as communicable at the risk factor level [4] and their effect increases in a multi-morbid situation.

In Ethiopia, hypertension and diabetes mellitus are among the leading causes of outpatient visits [2]. Among people under the age of 70 years deaths due to NCCDs are estimated to account for 37% of total deaths [5]. The common risk factors for NCCDS such as physical inactivity, inadequate intake of fruits and vegetables, alcohol consumption, cigarette smoking and overweight are widely prevalent in the country [2]. In the Hawassa town administration where the current study setting is located, 1517 cancer cases, 9209 diabetes mellitus cases, 13,420 cardiovascular disease cases and 3199 chronic obstructive pulmonary disease cases were reported in 2016 [6].

The co-existence of two or more NCCDs in an individual (multi-morbidity) is an emerging public health issue because of increasing in prevalence. Previous studies have reported a prevalence rate ranging from 17 to 90% [7–9]; the rates are higher among women [10–13] and among the elderly [11]. Because of aging of the population and longer survival, scientific advances in medical care that contributed to a longer survival, a proportion of population surviving longer with multiple NCCDs is growing, particularly in developing countries [14–16].

NCCDs co-occurrence is beyond chance, and have implication for public health [17]. People with multi-morbidity are heavier users of primary care services and polypharmacy [18, 19] and they are thought to be at increased risk of receiving sub-optimal care [20, 21]. Multi-morbidity may result in complex self-care needs [22]; it increases use of emergency facilities; and causes fragmented, costly, and ineffective care [23].

Having multiple NCCDs is associated with poor outcomes. Patients with multiple NCCDs have poor quality of life [24], mental health problems [25], more frequent and longer hospital stays [26, 27], more postoperative complications, a higher mortality and death at younger age, as well as impairments of physical and social functioning [26, 28, 29]. Moreover, there is an association between increasing number of NCCDs and disability [26, 28, 29].

Despite these consequences, studies on multi-morbidity are limited in developing countries. There are a few reports from the Middle East [13], Australia [7, 9, 17], Europe [11], the United States [12], and Canada [30], which estimated the prevalence of multi-morbidity. However, the reports showed huge variation. Due to improvements in the socioeconomic status and changes in the life style of people, NCCDs are becoming common public health problems in Ethiopia. Despite the burden and public health importance of the diseases, there is no report on prevalence of multi-morbidity in South Ethiopia. The aim of this study was to describe the prevalence of NCCDs and multi-morbidity among patients attended the outpatient department (OPD) of Hawassa University Comprehensive Specialized Hospital (HUCSH).

## Methods

### Study setting and design

A cross sectional study was conducted among patients attending at the HUCSH, OPD. Hawassa town is the capital of both the Sidama Zone and the Southern Nations and Nationalities Peoples Region (SNNPR). The town is located at 275 Km to the South of Addis Ababa with a total population of more than 500,000. HUCSH is the largest hospital in southern Ethiopia with more than 300 beds. The hospital was established in 2004 and it provides a comprehensive and specialized health services. The outpatient department consists of 5 units; namely medical OPD, surgical OPD, paediatrics OPD, Obstetrics and Gynaecology OPD and OPD for special clinics. The average number of patients flow at the OPD is about 200 patients per day.

### Study period and population

**Fig. 1:**
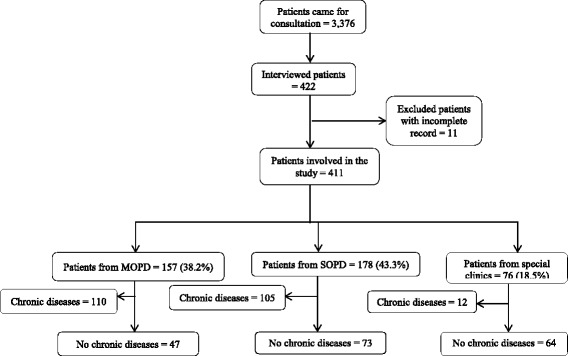
Number of patients selected from each outpatient department. MOPD: medical outpatient department; SOPD: surgical outpatient department; special clinics: ear-nose-throat clinic, dermatology unit and dental clinic

We did the study between January and February 2016. The study population was drawn from all patients attending the special clinics (ear-nose-throat clinics, dermatology unit, dental clinic), medical OPDs and surgical OPDs (Fig. 1). Patients who came for referral clinics were also involved. We included patients at least 18 years old; able to give informed consent and not seriously sick (not in severe pain). Patients unable to hear, unconscious or with serious mental disability and pregnant women were excluded from the study.

### Definitions

Chronic disease was defined as self-reported presence of non-infectious diseases diagnosed by physician, which produces non reversible pathological change and affecting selected systems of the body. Multi-morbidity was defined as the presence of two or more of the NCCDs by counting the self-reported presence of NCCDs in an individual [8, 11]. The following lists of diseases were identified and included in this report. Congestive heart failure (CHF), haemorrhoid, hypertension, rheumatic heart diseases, COPD (this includes asthma, chronic bronchitis and emphysema), liver diseases, benign prostatic hypertrophy (BPH), cholelithiasis, nephrolithiasis, nephritis (chronic kidney diseases), arthritis, gauty arthritis, diabetes mellitus, goiter, epilepsy, alzheimer disease, migraine and cancer of any organ.

### Variables

Multi-morbidity was taken as the main response variable. Having any of the NCCDs was a secondary dependent variable. Explanatory variables determining multi-morbidity and having of NCCDs were socio-demographic variables (sex, age, literacy status, monthly household income) and other variables like smoking, alcohol drinking, khat chewing, body mass index (BMI), lack of physical activity and low consumption of fruits and vegetables. Alcohol drinking was defined as past or current drinking of any alcoholic beverages in regular basis. In this study, khat chewing was defined as past or current chewing of khat (a psychoactive substance) in habitual basis. Patients with a history of past or current smoking any amount of cigarette in regular basis were considered as smokers. Recommended level of physical activity was defined as performing more than 20–30 min of moderate intensity exercise (for example hurried walking) for at least four times per week [31]. Patients who consume 125 ml (400 g) of fruits and vegetables per day were considered as having a recommended level of fruit and vegetable consumption [31].

### Sampling

The sample size required for this study was determined using the formula for estimating single population proportion [32].

$$ n=\frac{\mathrm{Z}{\left(\frac{\upalpha}{2}\right)}^2}{d^2}\mathrm{p}\left(1-\mathrm{p}\right) $$

Where, **n** is the sample size, **p** is the anticipated population proportion, **Z** is the level of significance (97% = 1.96) and **d** is the margin of error (0.05). We did not find study report on prevalence of multi-morbidity which used the population type and study design we considered. Therefore, we assumed a prevalence value of 0.5. Based on this, we calculated a sample size of 422 patients including the 10% non-response cases and interviewed all of them. However, 11 patients with missing values were excluded from analysis. Therefore, this report is based on 411 patients. Every 8th patient was interviewed in each room of the OPDs until the required sample size was obtained.

### Data collection

We developed a questionnaire format based on the objective of the study. Questions were first developed in English and translated into Amharic and then back translated to English. Each study participant was interviewed after consulting the physician. Presence of chronic diseases was assessed by a semi closed question “Do you have any of the following lists of chronic diseases diagnosed by a physician?”. Patients’ cards and clinical charts were reviewed to obtain additional information from the history, physical examination, investigations and the medications used. Drinking alcohol, smoking and khat chewing were assessed by asking questions if the patient had an experience of the behaviours in regular basis. We used similar type of questions to measure a recommended level of performing physical exercise and consumption of fruits and vegetables. Weight and height of patients were measured. Nurses who work at each OPD room collected the data. A supervisor with a bachelor degree in nursing was employed to coordinate the data collection.

The principal investigator provided training to data collectors and the supervisor. Training included explanation of the study objectives, explanation of each question and techniques for interviewing. A pilot study was conducted in HUCSH to determine the feasibility of the study protocol, to ensure comprehensiveness of the questionnaire items and to check if questions were clearly presented in a consistent manner and accurately addressed the research questions. The researchers agreed to include 20 adults for the pilot study. Supervision was done throughout the data collection process, and daily checking of the collected data was done in order to keep consistency and problems encountered were managed accordingly.

### Data processing and analysis

Data were entered and analysed using SPSS version 20 statistical packages (SPSS Inc., Chicago, IL, USA). BMI was calculated as weight in kilogram divided by height in meter square. The normal BMI considered in this study was 18.5–24.9 Kg/m^2^ [2]. Income was dichotomized in to two using the median monthly household income of 1500 Ethiopian Birr. The average exchange rate during the study period was 1 USD to 21.3673 Ethiopian Birr. Frequencies were calculated. Bivariate and multivariate logistic regression analyses were done to determine the relationship between dependent and independent variables. Variables with *p* value less than 0.2 in the bivariate logistic regression were included in the multivariate analysis. The strength of association between determinant variables and having of any of the NCCDs or multi-morbidity were measured through odds ratios.

### Ethics statement

The study protocol was reviewed and approved by the Hawassa University College of Medicine and Health Sciences Institutional Review Board (IRB). Ethical approval letter was written on 17/11/2015 and the reference number of the letter is Ref. No. IRB-019-08. Before the data collection, informed verbal consent was obtained from each study participant. The data were collected and analysed anonymously.

## Results

Among 411 patients’ involved in the study, 178 (43.3%) recruited from the surgical OPD (Fig. 1). Majority of the study participants, 234 (56.9%) were male, 158 (38.4%) were aged 18–29 years, 268 (65.2%) were urban dwellers, 101 (24.6%) were employed and 280 (68.1%) were married. Of the participants, 165 (40.1%) were non-educated. The mean (SD) age of the study participants was 37.6 (15.9) years. More than 55% of the patients (229 patients) had a monthly household income of above 1499 Ethiopian Birr (Additional file 1) and (Table 1).

**Table 1:** Socio-demographic characteristics of patients at HUCSH, February 2016

Characteristics	Value	Number	%
Address	Urban	268	65.2
	Rural	143	34.8
Sex	Male	234	56.9
	Female	177	43.1
Age in years	18–29	158	38,4
	30–44	119	29,0
	> 44	134	32,6
Occupation	Employed	101	24.6
	Farmer	99	24.1
	House wife	75	18.2
	Student	70	17.0
	Daily laborer	17	4.1
	Other	49	11.9
	Married	280	68.1
Marital status	Single	118	28.7
	Others^a^	13	3.2
Household income in Ethiopian Birr^a^	100–1499	182	44.3
	> 1499	229	55.7
Educational status	No education	165	40.1
	Literate	246	59.9

^a^Others = divorced and widowed, Exchange rate 1 USD to 21.3673 Ethiopian Birr

Majority (98.3%) of the patients ate vegetables in their daily meals. Alcohol drinking, khat chewing and cigarette smoking were experienced by 60 (14.6%), 41 (10.0%) and 11 (2.7%) patients, respectively. Concerning the BMI, 83 (20.2%) of the patients had a BMI score of at least 25 (Table 2).

**Table 2:** Unhealthy lifestyle factors among patients at HUCSH, February 2016

Factors	Yes; *n* (%)	No; *n* (%)
Exercising	274 (66.7)	137 (33.3)
Fruit and vegetable consumption	404 (98.3)	7 (1.7)
Alcohol drinking	60 (14.6)	351 (85.4)
Khat chewing	41 (10)	370 (90.0)
Smoking	11 (2.7)	400 (97.3)
BMI ⩾ 25	83 (20.2)	328 (79.8)

*BMI* body mass index, *n* number

More than 55 % (227 patients) were affected by at least one of the NCCDs. Musculoskeletal problems were the most frequent health conditions reported, 69 (16.8%). Diseases affecting the gastrointestinal system were the least of all health problems, 7 (1.7%). Overall, 73 (17.8%) of the patients had multi-morbidity (Table 3). The leading pairing of NCCDs was cardiovascular and endocrine system diseases, 10 pairs (Table 4).

**Table 3:** Distribution of chronic diseases by systems of the body among patients at HUCSH, February 2016

Body systems affected	Diseases	Yes	No
		n^*^ (%)	n^*^ (%)
Musculoskeletal system	Arthritis, Gauty arthritis	69 (16.8)	342 (83.2)
Endocrine system	Goiter, diabetes mellitus	67 (16.3)	344 (83.7)
Cardiovascular system^*^	HTN, CHF, RHD, hemorrhoid	60 (14.6)	351 (85.4)
Nervous system	Epilepsy, alzheimer, migraine	46 (11.2)	365 (88.8)
Genitourinary system^*^	BPH, nephrolithiasis, nephritis	30 (7.3)	381 (92.7)
Respiratory system^*^	COPD	19 (4.6)	392 (95.4)
Malignant cancer	Cancer	19 (4.6)	392 (95.4)
Gastrointestinal system	Liver diseases, cholelithiasis	6 (1.5)	405 (98.5)
Chronic diseases		227 (55.2)	184(44.8)
Multi-morbidity		73 (17.8)	338 (82.2)

^*^n number, *HTN* hypertension, *CHF* congestive heart failure, *RHD* Rheumatic heart disease, *BPH* benign prostatic hypertrophy, *COPD* chronic obstructive pulmonary diseases

**Table 4:** Chronic diseases pairing by systems of the body among patients at HUCSH, February 2016

Chronic disease pairing	Number	%
No chronic diseases	186	45.3
One chronic diseases	152	37.0
Cardiovascular and endocrine system diseases	10	2.4
Cardiovascular and musculoskeletal system diseases	5	1.2
Other pairs	38	9.3
Triple or more	20	4.8
Total	411	100

Factors such as age above 44 years (Adjusted odds ratio (AOR) = 2.7, 95% CI 1.5–4.8), household income of at least 1500 Ethiopian Birr (AOR = 1.6, 95% CI 1.0–2.5), no schooling (AOR = 1.7, 95% CI 1.0–2.7) and a BMI score of 25 or more (AOR = 2.0, 95% CI 1.1–3.7) predicted having of NCCDs (Table 5). Concerning multi-morbidity, in bivariate analysis, age, alcohol drinking, khat chewing, smoking and BMI showed an association with having of multi-morbidity. However, only age above 44 years (AOR = 4.4, 95% CI 2.2–8.8) maintained the significance in predicting multi-morbidity. Details on factors determining multi-morbidity are described in Table 6.

**Table 5:** Risk factors of having chronic disease among patients at HUCSH, February 2016

	NCCDs				
Variables	Yes	No	COR (95% CI)	AOR (95% CI)	*P*-value
Age in years					
18–29	65	93			
30–44	61	58	1.5 (0.9–2.4)	1.0 (0.6–1.7)	0.968
> 44	101	33	4.4 (2.6–7.3)	2.7 (1.5–4.8)	0.001
Household income in Ethiopian Birr					
100–1499	88	94			
> 1499	139	90	1.7 (1.1–2.5)	1.6 (1.0–2.5)	0.03
Education					
Literate	119	127			
No education	108	57	2.0 (1.4–3.0)	1.7 (1.0–2.7)	0.034
Exercise					
Yes	144	130			
No	83	54	1.4 (0.9–2.1)	1.2 (0.8–2.0)	0.384
Fruit and vegetable consumption					
No	6	1	5.0 (0.6–41.6)	4.8 (0.5–44.1)	0.166
Yes	221	183			
Khat chewing					
No	197	173			
Yes	30	11	2.4 (1.2–4.9)	1.6 (0.7–3.8)	0.287
Alcohol drinking					
No	185	166			
Yes	42	18	2.1 (1.2–3.8)	1.2 (0.6–2.5)	0.574
Cigarette smoking					
No	218	182			
Yes	9	2	3.8 (0.8–17.6)	1.9 (0.3–10.9)	0.462
BMI					
18.5–24.9	127	126			
< 18.5	39	36	1.1 (0.6–1.8)	1.2 (0.7–2.0)	0.624
⩾ 25	61	22	2.8 (1.6–4.8)	2.0 (1.1–3.7)	0.022

Exchange rate 1 USD to 21.3673 Ethiopian Birr

*NCCDs* non-communicable chronic diseases, *COR* crude odds ratio, *AOR* adjusted odds ratio, *95% CI* 95% confidence interval, *BMI* body mass index

**Table 6:** Risk factors of multi-morbidity among patients at HUCSH, February 2016

	Multi-morbidity				
Variables	Yes	No	COR (95% CI)	AOR (95% CI)	*P*-value
Age in years					
18–29	14	144			
30–44	15	104	1.5 (0.7–3.2)	1.3 (0.6–2.8)	0.559
> 44	44	90	5.0 (2.6–9.7)	4.4 (2.2–8.8)	0.000
Household income in Ethiopian Birr					
100–1499	26	156			
> 1499	47	182	1.6 (0.9–2.6)	1.2 (0.7–2.1)	0.608
Alcohol drinking					
No	55	296			
Yes	18	42	2.3 (1.2–4.3)	1.3 (0.6–2.9)	0.471
Khat chewing					
No	61	309			
Yes	12	29	2.1 (1.0–4.3)	1.3 (0.5–3.3)	0.602
Exercise					
Yes	55	219			
No	18	119	0.6 (0.3–1.1)	0.5 (0.3–1.0)	0.038
Smoking					
No	68	332	4.1 (1.2–13.7)	2.5 (0.6–10.4)	0.201
Yes	5	6			
BMI					
18.5–24.9	40	213			
< 18.5	9	66	0.7 (0.3–1.6)	0.8 (0.3–1.8)	0.547
⩾ 25	24	59	2.2 (1.2–3.9)	1.4 (0.7–2.7)	0.353

Exchange rate 1 USD to 21.3673 Ethiopian Birr

*COR* crude odds ratio, *AOR* adjusted odds ratio, *95% CI* 95% confidence interval, *BMI* body mass index

## Discussion

High prevalence of NCCDs and multi-morbidity was observed in this cross-sectional study. The predominant diseases that affected the patients were diseases of the musculoskeletal system. The risk of having any of the chronic diseases was highest among patients aged above 44 years and it was higher patients with a monthly household income over 1499 Ethiopian Birr, non-educated patients and patients whose BMI score was high. There was a highest risk of multi-morbidity among older patients.

The prevalence of NCCDs in the current study was higher than the report from Gilgelgibe (8.9%), Southwest Ethiopia [33]. The variation could be related to the differences in the study design, setting and the definition. The present study was an institution based cross-sectional study done among adult patients at a tertiary level health care facility and included 20 NCCDs. While in Gilgelgibe they did a community based survey in the general population among 15–64 age group and considered diseases like diabetes mellitus, cardiac diseases, hypertension, asthma, epilepsy and mental diseases [33].

In southwest Ethiopia, morbidities such as cardiac disease and hypertension were the leading health problems [34]. In Sweden, cardiovascular and mental diseases were the most common chronic disorders reported [11]. Diseases of the circulatory system and endocrine system were most prevalent in Greece [35]. Unlike the finding in these studies, chronic arthritis, hypertension and diabetes mellitus were the most common diseases affected the study participants in our study. The present study finding is in agreement with what has been reported in Bangladesh [13].

Multi-morbidity in the current study was lower than the report from rural Bangladesh [13], Canada [8] and Australia [7]. The prevalence was 53.8% in Bangladesh, 89% in Canada and 37.1% in Australia [7, 8, 13]. These differences could be partly explained by variations in the type of study population. The study in Bangladesh considered people aged above 60 years [13]. The studies in Canada and Australia were based on secondary data analysis and carried out among adults [7, 8].

According to the report by Calypse AB and colleagues, many patients suffered from multi-morbidity by chronic pain and arthritis [10]. In Australia, arthritis or chronic back pain and vascular diseases were the leading NCCDs combination [7]. Similar measure in the current study was cardiovascular diseases and diseases of the endocrine system. The adverse effect of NCCDs on both an individual patient and on the public is higher in a multi-morbid situation [17, 24–27]. Screening, timely diagnosis and management of NCCDs is important to limit the effects of multi-morbidity; therefore, particular attention should be given to the specific pairings as the co-occurrence may have some unidentified factors [17].

Studies in various settings reported the presence of strong association between advanced age and multi-morbidity [8–12]. In agreement with the findings of these studies, in the present study patients aged above 44 years had a higher risk of multi-morbidity. Also, higher age predicted an increased probability of having of an individual chronic disease. Interventions like identifying and addressing modifiable risk factors; screening for common NCCDs; treatment and follow-up of patients with common NCCDs could help in minimizing the burden and effects of increasing age on both acquiring an individual chronic disease and multi-morbidity [2].

A report from Sweden showed that multi-morbidity was higher among people with unhealthy behaviors [11]. In another study, life style factors such as high BMI predicted a risk of having of multi-morbidity [31]. In the current study, none of the life style factors predicted having of multi-morbidity. However, it supports the association between high BMI and the risk of having any of the NCCDs. We suggest the importance of taking actions targeted on unhealthy behaviors like controlling BMI to prevent NCCDs. Balanced diet and regular physical exercise are suggested, which may help in minimizing the problem.

Reports from other settings [10–13] showed that, multi-morbidity was higher among women. Authors of these studies explained the reasons for the observed gender disparity as survivor bias and due to the difference in vulnerability for co-occurring diseases by gender [10–13]. In contrary to these reports, we couldn’t observe significant association between gender and having of chronic disease or the association between gender and multi-morbidity. The finding in the present study is in agreement with the reports from Australia and South Africa [7, 36].

Some studies reported the presence of association between multi-morbidity and education [9, 37]. In another study, low education increased the risk of multi-morbidity among the elderly [11]. In contrary to these reports, in the current study educational status did not show association with multi-morbidity. However, people with low educational status had increased odds of having the NCCDs. Therefore, improving educational status of the society and providing health education to adult people with low educational status could help in preventing NCCDs.

The risk of multi-morbidity was higher among people with low household wealth in Canada [10]. In contrary to this, a report from South Africa [36] showed that people with high income had a higher risk of having multi-morbidity. In the current study however, we found people with high income had a higher risk of having the NCCDs but not multi-morbidity. The data in the current study shows that, among people with high income, about 90% had no education. If they have no knowledge of preventing these diseases, people with high income may experience unhealthy behaviors such as lack of exercise and low consumption of fruit and vegetables which in turn could be risk factors for developing the diseases [2]. Therefore, providing health education on how to prevent the NCCDs for peoples with high income could be important.

Some of the limitations of this study include firstly, the sample size was probably overrepresented by patients who came to a tertiary health facility because of multiple conditions. Secondly, the study design we used may be considered as one of the limitations in describing associations between range of factors and multi-morbidity. Analytic study designs could have been used to better determine the risk factors of multi-morbidity. Thirdly, excluding seriously sick cases from the study might have lowered the calculated estimate. Despite these limitations, the study findings generated valuable baseline information on the burden of NCCDs multi-morbidity and potential risk factors in the study area which could be tested in further studies.

## Conclusions

The present study finding suggests that there is a high prevalence of NCCDs and multi-morbidity among adult patients who visited the HUCSH. The NCCDs were particularly highest among the elderly and it was higher among people with high income, non-educated patients and among patients with high BMI. While multi-morbidity was highest among patients aged above 44 years. Primary-care strategy, including health promotion programs such as identifying and addressing modifiable risk factors; screening for common NCCDs; diagnosis, treatment and follow-up of patients with NCCDs and increasing educational opportunities are important to minimize the burden of NCCDs multi-morbidity.

## Additional file

Additional file 1: Dataset. Dataset supporting conclusions of the study on chronic diseases multi-morbidity among adult patients at Hawassa University Comprehensive Specialized Hospital, February 2016. (XLSX 73 kb)

## Abbreviations

BMI: Body mass index
BPH: Benign prostatic hypertrophy
CHF: Congestive heart failure
COPD: Chronic obstructive pulmonary diseases
HTN: Hypertension
HUCSH: Hawassa University Comprehensive Specialized Hospital
NCCDs: Non-communicable chronic diseases
OPD: Outpatient department
RHD: Rheumatic heart disease
SNNPR: Southern Nations and Nationalities Peoples Region

## References

[1] WHO (2014). Global status report on noncommunicable diseases: attaining the nine global noncommunicable diseases targets; a shared responsibility.

[2] EPHA (2012). Emerging public health problems in Ethiopia: chronic non-communicable diseases.

[3] Stuckler D (2008). Population causes and consequences of leading chronic diseases: a comparative analysis of prevailing explanations University of Cambridge. Milbank Q.

[4] Choi BCK, Bonita R, McQueen DV (2001). The need for global risk factor surveillance. J Epidemiol Community Health.

[5] WHO (2014). Non communicable diseases country profile.

[6] Hawassa Town Administration (2016). Hawassa town administration health department annual report.

[7] Britt HC, Harrison CM, Miller GC, Knox SA (2008). Prevalence and patterns of multimorbidity in Australia. Med J Aust.

[8] Fortin M, Bravo G, Hudon C, Vanasse A, Lapointe L (2005). Prevalence of multimorbidity among adults seen in family practice. Ann Fam Med.

[9] Taylor AW, Price K, Gill TK, Adams R, Pilkington R, Carrangis N, Shi Z, Wilson D (2010). Multimorbidity - not just an older person’s issue: results from an Australian biomedical study. BMC Public Health.

[10] Calypse AB, Darren L, Markus L, Tim C, Jeffrey JA (2012). Multimorbidity prevalence and patterns across socioeconomic determinants: a cross-sectional survey. BMC Public Health.

[11] Marengoni A, Winblad B, Karp A, Fratiglioni L (2008). Prevalence of chronic diseases and multimorbidity among the elderly population in Sweden. Am J Public Health.

[12] Agborsangaya CB, Ngwakongnwi E, Lahtinen M, Cooke T, Johnson JA (2013). Multimorbidity prevalence in the general population: the role of obesity in chronic disease clustering. BMC Public Health.

[13] Khanam MA, Streatfield PK, Kabir ZN, Qiu C, Cornelius C, Wahlin A (2011). Prevalence and patterns of multimorbidity among elderly people in rural Bangladesh: a cross-sectional study. J Health Popul Nutr.

[14] Valderas JM, Starfield B, Sibbald B, Salisbury C, Roland M (2009). Defining comorbidity: implications for understanding health and health services. Ann Fam Med.

[15] Kirchberger I, Meisinger C, Heier M, Zimmermann AK, Thorand B, Autenrieth CS, Peters A, Ladwig KH, Do A. Patterns of multimorbidity in the aged population: results from the KORA-age study. PLoS One. 2012;7:1. 10.1371/journal.pone.0030556 PMC3264590 22291986

[16] McCarron M, Swinburne J, Burke E, McGlinchey E, Carroll R, MCCallion P (2013). Patterns of multimorbidity in an older population of persons with an intellectual disability: results from the intellectual disability supplement to the Irish longitudinal study on aging (IDS-TILDA). Res Dev Disabil.

[17] Islam MM, Valderas JM, Yen L, Dawda P, Jowsey T, McRae IS (2014). Multimorbidity and comorbidity of chronic diseases among the senior Australians: prevalence and patterns. PLoS One.

[18] Agborsangaya CB, Lau D, Lahtinen M, Cooke T, Johnson JA (2013). Health-related quality of life and healthcare utilization in multimorbidity: results of a cross-sectional survey. Qual Life Res.

[19] Condelius A, Edberg AK, Jakobsson U, Hallberg IR (2008). Hospital admissions among people 65+ related to multimorbidity, municipal and outpatient care. Arch Gerontol Geriatr.

[20] Bayliss EF, Edwards AE, Steiner JF, Main DS (2008). Processes of care desired by elderly patients with multimorbidities. Fam Pract.

[21] Vogeli C, Shields AE, Lee TB, Gibson TB, Marder WD, Weiss KB, Blumenthal D (2007). Multiple chronic conditions: prevalence, health consequences, and implications for quality, care management, and costs. J Gen Intern Med.

[22] Wolff JL, Starfield B, Anderson G (2002). Prevalence, expenditures, and complications of multiple chronic conditions in elderly. Arch Intern Med.

[23] Fortin M, Soubhi H, Hudon C, Bayliss EA, Akker MVD (2007). Multimorbidity’s many challenges. BMJ.

[24] Fortin M, Bravo G, Hudon C, Lapointe L, Almirall J, Dubois MF, Vanasse A (2006). Relationship between multimorbidity and health-related quality of life of patients in primary care. Qual Life Res.

[25] Fortin M, Bravo G, Hudon C, Lapointe L, Dubois MF, Almirall J (2006). Relationship between psychological distress and multimorbidity of patients in family practice. Ann Fam Med.

[26] Bayliss EA, Bayliss MS, Ware JE, Steiner JF (2004). Predicting declines in physical function in persons with multiple chronic medical conditions: what we can learn from the medical problem list. Health Qual Life Outcomes.

[27] Byles JE, D’Este C, Parkinson L, O’Connell R, Treloar C (2005). Single index of multimorbidity did not predict multiple outcomes. J Clin Epidemiol.

[28] Marengoni A, Von Strauss E, Rizzato D, Winblad B, Fratiglioni L. The impact of chronic multimorbidity and disability on functional decline and survival in elderly persons. A community-based, longitudinal study. J Intern Med. 2009;265:288–95. 10.1111/j.1365-2796.2008.02017.x 19192038

[29] Loza E, Jover JA, Rodriguez L, Carmona L. Multimorbidity: prevalence, effect on quality of life and daily functioning, and variation of this effect when one condition is a rheumatic disease. Semin Arthritis Rheum. 2009;38:312–9. 10.1016/j.semarthrit.2008.01.004 18336872

[30] Fortin M, Lapointe L, Hudon C, Vanasse A (2005). Multimorbidity in medical literature: is it commonly researched?. Can Fam Physician.

[31] Fortin M, Jeannie H, José A, Tarek B, Maxime S, Martin L (2014). Lifestyle factors and multimorbidity: a cross sectional study. BMC Public Health.

[32] Daniel WW (2010). Biostatstics: basic concepts and methodology for the health sciences.

[33] Ayalew MT, Abraham H, Fasil T, Fessahaye A, Kifle W, Makonnen A, Yoseph M, Solomon T, Gemeda A, Amare D, et al. Population based survey of chronic noncommunicable diseases at Gilgelgibe field research center, southwest Ethiopia. Ethiop J Health Sci. 2012;22(Special Issue):7–18.

[34] Martin P. Chronic non-communicable diseases in Ethiopia - a hidden burden. Ethiop J Health Sci. 2012;22(2). PMC3542741 23319834

[35] Minas M, Koukosias N, Zintzaras E, Kostikas K, Gourgoulianis KI. Prevalence of chronic diseases and morbidity in primary health care in central Greece: an epidemiological study. BMC Health Serv Res. 2010;10(252). 10.1186/1472-6963-10-252 PMC2939599 20799979

[36] Olufunke A, Lumbwe C (2013). The social determinants of multimorbidity in South Africa. Int J Equity Health.

[37] Gabriele N, Richard P, Stefanie B, Silke H, Sabine R, Jakob L (2008). The impact of education on risk factors and the occurrence of multimorbidity in the EPIC-Heidelberg cohort. BMC Public Health.

